# Cuckoo Hosts Fine‐Tune Their Egg Rejection After Experiencing a Parasitism Event

**DOI:** 10.1002/ece3.70825

**Published:** 2025-01-07

**Authors:** Bin Li, Longwu Wang, Jianping Liu, Wei Liang

**Affiliations:** ^1^ College of Biological Sciences and Engineering North Minzu University Yinchuan China; ^2^ Ministry of Education Key Laboratory for Ecology of Tropical Islands, Key Laboratory of Tropical Animal and Plant Ecology of Hainan Province, College of Life Sciences Hainan Normal University Haikou China

**Keywords:** brood parasitism risk, optimal acceptance threshold, recognition mechanism, *Saxicola ferreus*

## Abstract

Recognising and rejecting parasitic eggs is one of the most common anti‐parasitism strategies used by host birds. However, the egg rejection of some hosts exhibits behavioural plasticity. To investigate whether the egg rejection behaviour of host birds changes after encountering a parasitism event, we conducted egg rejection experiments on the locally most common host of the common cuckoo ( *Cuculus canorus* ), the grey bushchat ( *Saxicola ferreus* ) in Yunnan, China. When a single pure white egg of the white‐rumped munia ( *Lonchura striata* ) or a blue model egg was individually added to the nest of the grey bushchat, the rejection rate for the white‐rumped munia eggs was as high as 93.3%, whereas the rejection rate for the blue model egg was minimal (5.56%). However, when the grey bushchat rejected the munia egg and a blue model egg was subsequently added to its nest, the rejection rate for the blue model egg was significantly higher, reaching 54.5%. When recognised, the presence of a non‐mimetic foreign egg in the nest may then act as a cue indicating high parasitism risk for the host. Consequently, the bird may intensify its inspection of nest eggs, leading to increased rejection of the previously accepted blue model eggs. Our results are consistent with the optimal acceptance threshold hypothesis, suggesting that as the parasitism risk increases, the grey bushchat adjusts its acceptance threshold for foreign eggs to become more stringent.

## Graphical abstract

To investigate whether the egg rejection behaviour of host birds changes after encountering a parasitism event, we conducted egg recognition experiments in a population of the grey bushchat ( *Saxicola ferrea* ). The presence of the munia egg in the nest may serve as a cue of parasitism risk for the grey bushchat. As the predictability of parasitism risk increases, the grey bushchat adjusts its acceptance threshold for foreign eggs to become more stringent.

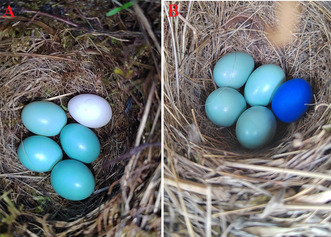

## 1 Introduction

Obligate brood parasitic birds do not build nests themselves. Instead, they lay their eggs in the nests of other birds (hosts), relying on the hosts to incubate the eggs and care for the chicks. This transfer of parental care costs to the host bird is well‐documented, and brood parasitism has evolved independently in seven different avian lineages (Davies and MdeL 1989; Davies 2000; Sorenson and Payne 2002; Soler 2014, 2017). Brood parasitism affects host species to different degrees, from not affecting reproductive output at all to imposing very high reproductive costs (Payne 1977; Davies 2000; Langmore et al. 2005; Medina and Langmore 2015). In turn, this has led to different selective pressures that have resulted in different types of nest defence at several stages (Davies 2011; Feeney, Welbergen, and Langmore 2012; Soler 2014; Dillenseger 2024). Some hosts reduce the risk of parasitism by selecting their nesting sites (Forsman and Martin 2009; Tolvanen, Forsman, and Thomson 2017), for example, by relying on humans to provide shelter and thus reducing the risk of being parasitised, so they build nests close to or directly in human habitats (Liang et al. 2013; Zhang et al. 2023). Some host birds defend against parasites by driving away and attacking approaching brood parasites (e.g., the common cuckoo, *Cuculus canorus* ) (Davies and Welbergen 2009; Zhao et al. 2022; but see Yang et al. 2014) or by using passive defence, such as nest concealment, inhibitory or deceptive nest structures, or phenological mismatch to reduce the likelihood of parasitism (Grim et al. 2011; Feeney, Welbergen, and Langmore 2012; Li et al. 2016; Medina and Langmore 2016). On the other hand, some host birds are capable of recognising and rejecting parasitic chicks to minimise the costs of mistakenly caring for non‐offsprings (Langmore, Hunt, and Kilner 2003; Grim, Kleven, and Mikulica 2003; Sato et al. 2010; Noh, Gloag, and Langmore 2018; Arco et al. 2023). Among all defences, host recognition and rejection of parasite eggs are the most common and crucial anti‐parasitic strategies (Ruiz‐Raya and Soler 2020; Ye et al. 2022; Liu, Wang, and Liang 2023; Zhang et al. 2023a; Dillenseger 2024). This stage represents an optimal opportunity to prevent brood parasitism as hosts have not yet heavily invested in the given breeding attempt.

However, hosts with egg recognition abilities do not exhibit absolute rejection of foreign parasitic eggs (Hauber, Moskát, and Bán 2006). In fact, the variability in behaviour associated with rejection has been confirmed by many studies. Firstly, the host shows variability in its rejection responses. For example, when the host recognises the parasitic egg, its response includes grasping and ejecting it from the nest, puncturing it, burying it, or abandoning the nest (Davies and MdeL 1989; Moskát and Honza 2002; Guigueno and Sealy 2010; Stokke et al. 2010). Alternatively, it may choose to accept the foreign egg (Antonov et al. 2009; Soler et al. 2012; Ruiz‐Raya et al. 2015). Secondly, the host's recognition threshold can show variability (Hauber, Moskát, and Bán 2006; Moskát and Hauber 2007; Ruiz‐Raya and Soler 2020).

The optimal acceptability threshold hypothesis predicts animals can make flexible adjustments to their individual acceptance thresholds in order to respond to variable environmental contexts (Reeve 1989). Research in brood parasitism‐host systems does indeed show that the host can flexibly adjust its response to parasitic eggs. Studies have shown that factors including different stages of the reproductive cycle (e.g., Moskát and Hauber 2007), host age and experience (Moskát, Bán, and Hauber 2014), the cost of recognising and rejecting parasitised eggs (Davies, MdeL, and Kacelnik 1996), egg colouration (Polačiková and Grim 2010; Hauber et al. 2014), egg size (Soler et al. 2015), egg mass (Ruiz‐Raya et al. 2015) and the risk of nest parasitism (Lindholm 2000; Thorogood and Davies 2013, 2016; Zhang et al. 2021, 2022) can all influence host egg rejection decisions. The host can benefit from optimising the trade‐off between the fitness gains derived from the advantages of egg rejection and the costs associated with mistakenly rejecting its own eggs by adjusting thresholds flexibly (Stokke et al. 2002; Ruiz‐Raya and Soler 2020). If the acceptance threshold of the host is too permissive, the probability of acceptance errors will increase; whereas if the threshold is too restrictive, the probability of recognition errors will increase (Reeve 1989). It can be predicted that hosts will adjust their acceptance threshold according to the environmental context, parasite risk increases and the threshold will become more restrictive (Liebert and Starks 2004; Ruiz‐Raya and Soler 2020).

Some studies have shown that host birds can adjust their egg rejection decisions based on perceived parasitism risk (Lindholm 2000; Moksnes, Hagen, and Honza 2000; Thorogood and Davies 2013, 2016; Feeney et al. 2015; Thorogood and Davies 2016; but see Štětková et al. 2023). For example, despite not being parasitised, the red‐whiskered bulbul ( *Pycnonotus jocosus* ) adjusts its sensitivity to parasite eggs by evaluating the parasitic timing of the cuckoos, and it exhibits a significantly higher rejection rate of foreign eggs during the egg‐laying period compared to the incubation period (Liu et al. 2021). In the breeding area of the Daurian redstart ( *Phoenicurus auroreus* ), placing specimens of the common cuckoo and playing the calls of male cuckoos to simulate the presence of the brood parasite and an increased risk of parasitism significantly increased the rejection rate of foreign eggs by the redstart (Zhang et al. 2022). Similarly, the same experiment done in proximity to nests of great reed warblers ( *Acrocephalus arundinaceus* ) also increased the rejection rate of experimental parasitic eggs (Bartol et al. 2002). Although these studies involved hosts from different continents that were parasitised by different cuckoo species or different common cuckoo lineages, they all seem to suggest that the presence of parasites near the host's nest increases the likelihood of egg rejection by the host. However, the sighting of a cuckoo in a breeding area or near a nest may not be a sufficiently reliable clue for parasitism to hosts. This is because not every common cuckoo visit leads to a brood parasitism event (Štětková et al. 2023). Previous studies focused on the male cuckoo, while the female actually parasitises nests and adopts a secretive behaviour and rapid laying (Davies 2011). Thus, previous experiments did not replicate conditions observed in the wild. While those aforementioned studies are interesting, they may not encapsulate those brood parasitism events where the brood parasite is not detected. The only evidence would be the egg. However, we have little evidence to support the optimal acceptability threshold hypothesis. Therefore, we conducted a study on the egg rejection behaviour of the grey bushchat ( *Saxicola ferreus* ) using white‐rumped munia eggs ( *Lonchura striata* ) and blue model eggs. The gray bushchat is the locally most common host of the common cuckoo. They both lay blue eggs, which look very similar to each other in human eyes. We have no record of grey bushchats rejecting cuckoo eggs, but Zhong et al. (2023) indicated that some grey bushchats parasitised by cuckoos will abandon their nests. We utilised the highly non‐mimetic pure white eggs of the white‐rumped munia and blue model eggs to simulate parasitic events and observed the responses of the grey bushchat to the low mimetic blue model eggs after experiencing a previous brood parasitism event. We hypothesised that egg rejection by grey bushchats is greatly influenced by prior exposure to brood parasitism.

## 2 Materials and Methods

### 2.1 Study Area and Study Species

**FIGURE 1:**
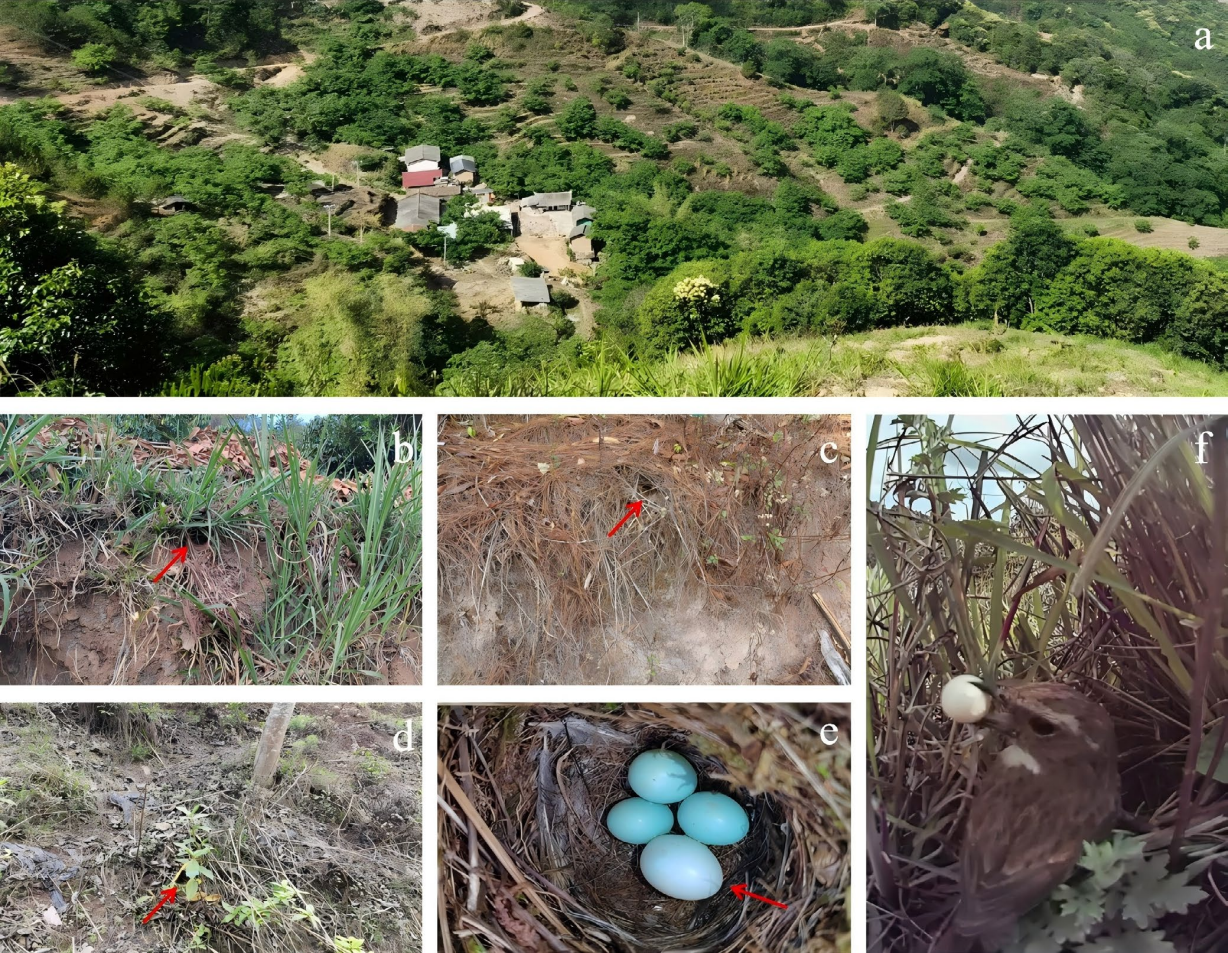
Landscape of the study site (a), various breeding habitats and nest sites of the grey bushchat [nesting on barren slopes (b‐c), near the grassroots (d)], the eggs of the cuckoo and grey bushchat (e) and the egg rejection process of the grey bushchat (f). The arrows are pointing at the location of the nest and cuckoo egg.

Jingdong County (23°56′ ~ 24°29′ N, 100°22′ ~ 101°15′ E) is located in the central‐southern part of Yunnan, southwestern China. Our research site, Caihu Village, was located in the southeast of Jingdong County. It has an elevation of 1767 m, the annual mean temperature is 15°C and the annual precipitation of 1500 mm (Liu, Li, and Liang 2024). The study site is situated in a sparsely inhabited mountainous region, predominantly encompassing natural secondary forests and artificial forests (Figure 1a; Liu, Li, and Liang 2024).

The grey bushchat is a small passerine bird belonging to the Muscicapidae family. It is one of the hosts of the common cuckoo (Yang et al. 2012; Zhong et al. 2023). At our study site, the grey bushchat breeds from early April to late July, nesting on cultivated land (Figure 1b), barren slopes (Figure 1c) and near grassroots (Figure 1d,f) in scrub forests with relatively secluded nests. Females typically lay blue eggs (Figure 1e), with a clutch size of 3–6 eggs. They begin incubating the eggs when the clutch is full, and the incubation period is approximately 12 days. At our study site, we monitor about 150 nests of the grey bushchat annually, with a parasitism rate of 13%–26%. The common cuckoo also lays blue eggs (Figure 1e).

### 2.2 Egg Recognition Experiments

**FIGURE 2:**
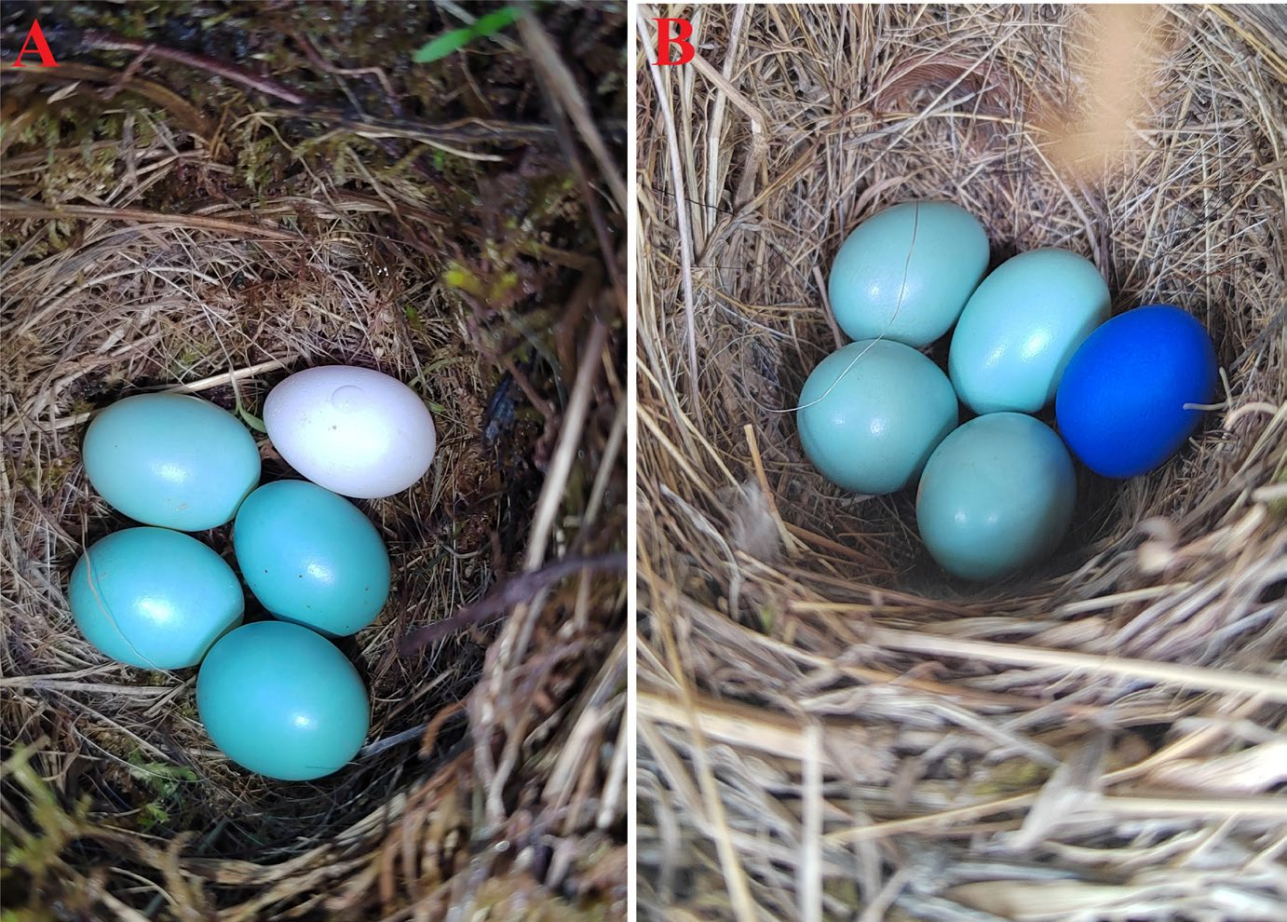
Egg recognition treatments in nests of the grey bushchat. (A) Adding one white‐rumped munia egg to the nest; (B) adding one blue model egg to the nest.

We systematically searched for active nests of the grey bushchat in the study area during the breeding season in April–July 2023. Due to the incubation period being approximately 12 days, we defined the first 3 days after clutch completion as the early stages of incubation. Nests of the grey bushchat during the early stages of egg incubation were randomly subjected to four treatments. Treatment 1 served as the control group, and no experimental eggs were added (*n* = 12). Regular nest checks were conducted every 2 to 3 days. Treatment 2 involved adding one white munia egg (Figure 2) to the grey bushchat nest (*n* = 15). The munia eggs were purchased as a commercially unfertilised egg from an online platform. For Treatment 3, one blue model egg (Figure 2) was added to the grey bushchat nest (*n* = 18). For Treatment 4, a group received first a munia egg, and after rejecting it, one blue egg was added (*n* = 11). The blue model eggs were made following method by Zhong et al. (2023), using plastic soft clay, to have similar size and mass compared to real bushchat eggs (the mean size of 8 blue model eggs of 17.5 mm × 13.3 mm compared with 17.6 mm × 13.8 mm of 130 grey bushchat eggs). The size of munia eggs (16.0 mm × 12.2 mm, *n* = 8) was slightly smaller than that of grey bushchat eggs. For nests in Treatment 2 and 3, we added one experimental egg to the nest immediately after the full clutch of eggs was laid, that is, on the first day of incubation, and checked the nest the next day, followed by checking it every 2–3 days thereafter. We monitored nests in groups Treatment 2 and 3 for 6 days. For nests in Treatment 4, we added one munia egg to the nest on the first day of the incubation period, after the grey bushchat rejected the munia egg and the blue model egg was directly added. After adding one munia egg to the nest, we checked the nest the following day and then checked it every 2–3 days thereafter. For munia eggs, 60% of the individuals would reject the eggs within 3 days. After adding another blue model egg to the nest, we continued to monitor for 6 days. Nests that were predated or artificially destroyed within the 6 days were excluded from the experimental results (Ye et al. 2022).

### 2.3 Data Analysis

Statistical analysis was conducted using IBM SPSS Statistics for Windows, Version 22.0 (IBM Corp., Released 2013, Armonk, NY: IBM Corp.). Fisher's exact test was used to compare the differences in egg discrimination behaviour among the experimental egg treatment types.

## 3 Results

**FIGURE 3:**
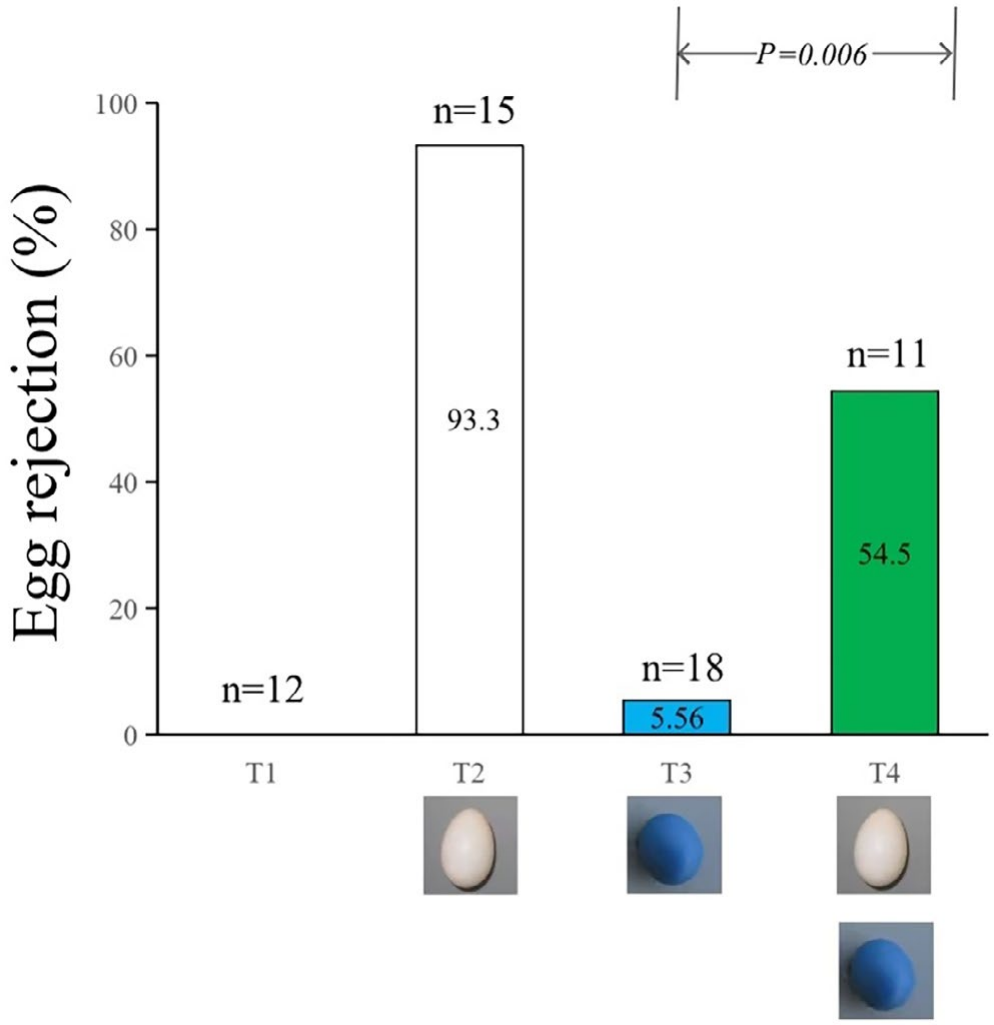
Egg rejection rates of the grey bushchat in the four treatments (T1 refers to Treatment 1) serves as the control group, and no experimental eggs were added, with regular nest checks every 2–3 days; T2 refers to Treatment 2, involving adding one white munia egg to the grey bushchat nest; T3 refers to Treatment 3, adding one blue model egg to the grey bushchat nest; and T4 refers to Treatment 4, adding one blue model egg after the grey bushchat rejected the munia egg.

The experimental results indicated that regular nest checks did not lead to the grey bushchat rejecting its own eggs or abandoning the nest. The grey bushchat tended to reject the munia egg within 1–2 days, with a rejection rate as high as 93.3%. The rejection rate for individually added blue model eggs to the nest was only 5.56%. In Treatment 4, the rejection rate of the grey bushchat towards the munia egg was 100%, and towards the blue model egg was 54.5%, which was significantly higher than that of Treatment 3 (Fisher's exact test, *x* ^*2*^ = 8.949, df = 1, *p* = 0.006; Figure 3).

## 4 Discussion

In this study, the grey bushchat population exhibited a high ability to recognise the highly non‐mimetic munia eggs. In a single parasitism treatment, grey bushchats largely did not recognise or reject the low‐mimetic blue model eggs. However, when previous parasitism events were simulated using non‐mimetic white‐rumped munia eggs, most grey bushchat individuals rejected the blue model eggs. This suggests that grey bushchats exhibited plasticity in their rejection behaviour towards eggs with similar phenotypic traits after experiencing a previous parasitism event. Our results are consistent with the optimal acceptance threshold hypothesis, where hosts flexibly adjust their acceptance thresholds for parasitic eggs in response to different cognitive scenarios, and after prior exposure to brood parasitism events, the host's acceptance threshold for foreign eggs becomes stricter (Reeve 1989).

Identifying and rejecting parasitic eggs allows hosts to mitigate the costs of brood parasitism and maximise their fitness (Davies 2000). However, host responses to parasitised eggs may also vary depending on differences in the costs associated with rejecting parasite eggs, the host's perceived risk of parasitism or the actual parasitism risk encountered (Davies, MdeL, and Kacelnik 1996; Thorogood and Davies 2013). For example, when a parasitism event occurs during the middle or late incubation period of the host, the parasite eggs may not receive sufficient incubation, resulting in their inability to hatch normally. In such cases, the cost of accepting the parasitic egg may be low whereas the cost of rejecting it may be high (i.e., in the process of rejecting parasite eggs, a bird may also puncture its own eggs), so hosts are likely to accept the parasitic eggs (Davies, MdeL, and Kacelnik 1996; Peer, McCleery, and Jensen 2018). In this study, for simulated parasitism of Treatment 3, the grey bushchats were in the early stage of the egg incubation period. Rejecting parasitic eggs at this stage would be highly adaptive, yet the birds essentially did not reject them. In contrast, in Treatment 4, when the blue model eggs were added to the nests of grey bushchats, they were already in the middle of the incubation period. At this time, accepting parasitic eggs would not incur significantly higher costs of parasitism, and there would be a low acceptance cost, making acceptance more adaptive. However, many of the birds rejected the parasitic eggs. This may be attributed to grey bushchats having more time to form a ‘template’ for their eggs, allowing them to compare them with dissimilar eggs. Although the recognition template can be inherited and/or learned from the first breeding attempts (Rothstein 1978; Lotem, Nakamura, and Zahavi 1995; Stokke et al. 2004, 2007; Wang et al. 2015), it can also be acquired again and updated with each new attempt (Soler et al. 2013). When the grey bushchats reacted to the parasitism event and rejected non‐mimetic munia eggs, they would carefully inspect the eggs in the nest. Therefore, in Treatment 4, the rejection rate of the grey bushchats towards the blue model eggs significantly increased. Some studies suggested that the cost of rejecting eggs may force some hosts to accept foreign eggs (Davies, MdeL, and Kacelnik 1996; Peer, McCleery, and Jensen 2018). In our Treatment 3, the grey bushchats accepted blue model eggs, while in Treatment 4, most bushchats rejected the blue model eggs. Breeding experience cannot explain this phenomenon, as it is unlikely that all birds in Treatments 2 and 3 were naïve and that those in Treatment 4 were experienced. Furthermore, it cannot be the case that the high cost is due to rejecting eggs. Since the munia eggs and model eggs used in the experiment were generally smaller than the grey bushchat eggs and smaller than the model eggs and budgerigar ( *Melopsittacus undulatus* ) eggs used by Zhong et al. (2023), the grey bushchats were able to reject the experimental eggs by grasping and removing them (Zhong et al. 2023). In this study, the grey bushchats indeed rejected the parasitic eggs by grasping and removing them, without abandoning the nest or incurring apparent rejection costs during the rejection process. The rejection costs may be only associated with the risk of misidentification of own eggs. Therefore, the costs of parasitism or rejection do not seem to explain the results of this study.

Several studies have argued for behavioural plasticity in host egg rejection (Bartol et al. 2002; Feeney et al. 2015; Thorogood and Davies 2016; Zhang et al. 2021, 2022), whereby hosts can flexibly adapt their egg rejection behaviours in response to their perceived risk of parasitism. Brief visits by parasites to the nest or their presence in the breeding area may serve as cues for parasitism risk, prompting hosts to increase their rejection of parasitic eggs (Bartol et al. 2002; Zhang et al. 2022). In this study, we did not manipulate or alter the parasitism risk in the environment. Instead, we experimentally parasitised the nests of the grey bushchat. When the grey bushchats reacted to the parasitism event, by recognising and rejecting non‐mimetic egg (indicated by the presence of non‐mimetic white‐rumped munia eggs in the nest, followed by rejection), it rejected eggs of the type that was in general accepted for individuals that did not reject a previous egg. The results of this study are consistent with those of Hauber, Moskát, and Bán (2006) for great reed warblers. They found that greater reed warblers that were exposed to (and rejected) non‐mimetic eggs experimentally added to their nests rejected manipulated eggs added to their nests at a much higher rate. However, naïve hosts with no experience of being exposed to parasitic eggs usually accepted manipulated eggs experimentally added to their nests. We suggest that artificially modifying the parasitism rate modifies the bushchats's perception of parasitism risk. The findings of the present study, like those of Hauber, Moskát, and Bán (2006) and Feng, Yang, and Liang (2019), support the optimal acceptance threshold hypothesis, that is, when the perceived risk of parasitism increases with the addition of experimental eggs, the acceptance threshold of grey bushchat for parasitised eggs becomes more stringent (Reeve 1989; Hauber, Moskát, and Bán 2006; Hanley et al. 2019; Ruiz‐Raya and Soler 2020).

In conclusion, our findings suggest that the grey bushchat, one of the common cuckoo hosts, may adjust its egg rejection decision after a parasitism event. Since we did not measure the colour of the experimental and grey bushchat eggs, it is unclear whether the blue model or munia eggs more closely resemble the colour of the grey bushchat eggs, since both are non‐mimetic to the human eye. It remains unknown why the grey bushchat tends to accept the blue model eggs and reject the munia eggs. Further research is needed to validate and understand this behaviour. In fact, the eggs laid by cuckoos are highly similar to the host eggs in background colour. However, for this study, non‐mimetic eggs were used, which do not represent real conditions of parasitism. Nevertheless, since the egg recognition ability of the grey bushchat population in this study has not been reported, it is necessary to use non‐mimetic eggs for the experiment. In the future, using mimetic eggs for the experiment will be closer to actual conditions and will help us understand how the grey bushchat's egg rejection behaviour is adjusted under high parasitism risk. Furthermore, the sample size of grey bushchats that accepted munia eggs was not large enough to see if this affected their acceptance of a subsequent blue egg. This treatment group would have complemented Treatment 4 and is essential for controlling variations in the incubation stage.

## Author Contributions

**Bin Li:** investigation (lead), methodology (equal), validation (equal). **Longwu Wang:** funding acquisition (equal), resources (equal), validation (equal), writing – review and editing (equal). **Jianping Liu:** conceptualization (equal), formal analysis (lead), resources (equal), supervision (equal), writing – original draft (lead), writing – review and editing (equal). **Wei Liang:** conceptualization (equal), funding acquisition (equal), resources (equal), supervision (equal), writing – review and editing (equal).

## Ethics Statement

The experiments comply with the current laws of China, where they were performed. Experimental procedures were in agreement with the Animal Research Ethics Committee of the Hainan Provincial Education Centre for Ecology and Environment, Hainan Normal University (No. HNECEE‐2022‐002).

## Conflicts of Interest

The authors declare no conflicts of interest.

## Supporting information


**Table S1.** Data of egg experiments in the grey bushchat ( *Saxicola ferreus* ) used for this study.

## Data Availability

Data used for this study are provided as Supporting Information (Table S1) and can be found online at https://figshare.com/s/2292f4993c9f3a91f6d2 (doi: https://doi.org/10.6084/m9.figshare.26976520).
